# Supplementary material for: Anomalous and Chern topological waves in hyperbolic networks
Source: Nat Commun. 2024 Mar 14;15:2293. doi: 10.1038/s41467-024-46551-x (PMC10937626; doi:10.1038/s41467-024-46551-x)
Supplement: Supplementary file 1 — Supplementary information [file 41467_2024_46551_MOESM1_ESM.pdf]

Supplementary Information for Anomalous and Chern topological waves in hyperbolic networks

Qiaolu Chen[1,2], Zhe Zhang[1], Haoye Qin[1], Aleksi Bossart[1], Yihao Yang[2], Hongsheng Chen[2], and Romain Fleury[1*]

[1] *Laboratory of Wave Engineering, School of Electrical Engineering, EPFL, Lausanne, Switzerland.*
[2] *Interdisciplinary Center for Quantum Information, State Key Laboratory of Modern Optical Instrumentation, ZJU-Hangzhou Global Science and Technology Innovation Center, College of Information Science and Electronic Engineering, ZJU-UIUC Institute, Zhejiang University, Hangzhou, China.*
[*] Corresponding author. Email: romain.fleury@epfl.ch
These authors contributed equally: Qiaolu Chen, Zhe Zhang

February 24, 2024

Supplementary Note 1: Model of a three-port circulator

The three-port circulator can be described by a unitary, C_3-symmetric scattering matrix S_0 [1–3],

$$S_0 = \begin{bmatrix} R & T & D \\ D & R & T \\ T & D & R \end{bmatrix}, \tag{1}$$

where

$$\begin{cases} R = -1 + \dfrac{2}{3}\cos\xi e^{i\xi} + \dfrac{2}{3}\cos\eta e^{i\eta}, \\ T = \dfrac{2}{3}\left[e^{-i\frac{2}{3}\pi}\cos\xi e^{i\xi} + e^{i\frac{2}{3}\pi}\cos\eta e^{i\eta}\right], \\ D = \dfrac{2}{3}\left[e^{i\frac{2}{3}\pi}\cos\xi e^{i\xi} + e^{-i\frac{2}{3}\pi}\cos\eta e^{i\eta}\right]. \end{cases} \tag{2}$$

The scattering matrix S_0 is therefore parameterized by two angular parameters ξ and η. These two angular parameters are transformed from the eigenvalues of right- and left-handed

eigenmodes of the circulator (i.e., ω_+ and ω_-), respectively, which are written as [2],

$$\begin{cases} \xi = \arctan(\dfrac{\omega - \omega_+}{\gamma}), \\ \eta = \arctan(\dfrac{\omega - \omega_-}{\gamma}). \end{cases} \tag{3}$$

Here, γ is the inverse of the delay time for three ports connecting to outer links, and ω is the excitation angular frequency. According to coupled-mode theory, for circulators built from Zeeman splitting, the parameters ξ and η determine both the amount of Zeeman splitting and the deviation of the excitation angular frequency ω from the right- and left-handed eigenvalues of the circulator ω_+ and ω_-. In other words, ξ and η are frequency-dependent parameters that control the non-reciprocal level of the circulator, with $\xi = \eta$ corresponding to reciprocal case, and $\xi = -\eta$ to excitation at $(\omega_+ - \omega_-)/2$.

Supplementary Note 2: Unit cell, Bravais lattice, and Floquet band structure of $\{8, 3\}$ hyperbolic lattice

According to the crystallography of hyperbolic lattices [4], the $\{8, 3\}$ hyperbolic lattice can be described by a $\{8, 8\}$ hyperbolic Bravais lattice, along with a reference unit cell taking the form of a regular octagon with 16 lattice sites. The four pairwise edges of a unit cell define four translation generators: $\gamma_\mu, \mu \in \{1, ..., 4\}$. It means that one octagon in $\{8, 8\}$ hyperbolic Bravais lattice can translate to another one by applying a product of the four generators and their inverses. In other words, the Bloch wave carries a phase factor e^{ik_μ} from one unit cell to the next.

By replacing the lattice sites with non-reciprocal three-port circulators, we obtain the unit cell of our $\{8, 3\}$ hyperbolic scattering network, as shown in Supplementary Fig.1b. The adjacent circulators inside a unit cell are connected by bidirectional links imparting a phase delay φ. The state vectors $|a_n\rangle$ and $|b_n\rangle$ denote the scattering wave amplitudes propagating out and into the circulators, respectively. Therefore, the scattering process of the 16 circulators in a unit cell can be characterized by

$$S_{\text{unitcell}}|b_n\rangle = S_{\text{element}} P_0 |b_n\rangle = |a_n\rangle, \tag{4}$$

where $S_{\text{element}} = \text{diag}(S_0, ..., S_0)$ contains scattering matrices of 16 circulators. The state vectors $|a_n\rangle$ and $|b_n\rangle$ are placed in order from 1 to 48, and P_0 is a 48×48 unitary permutation matrix leading the interchange of the order in $|b_n\rangle$. With the hyperbolic Bloch theory, we transfer the Eq.(4) into the momentum space as,

$$S_{\text{unitcell}}|b(\mathbf{k})\rangle = |a(\mathbf{k})\rangle. \tag{5}$$

Then we form a relation between $|a(\mathbf{k})\rangle$ and $|b(\mathbf{k})\rangle$ by adding a bidirectional phase delay φ and a Bloch phase delay $\pm k_\mu$, written as

$$|a(\mathbf{k})\rangle = e^{-i\varphi(\mathbf{k})}\Lambda(\mathbf{k})|b(\mathbf{k})\rangle, \tag{6}$$

where $\Lambda(\mathbf{k})$ is a diagonal matrix with $\Lambda_{27} = \Lambda_{47} = e^{-ik_1}$, $\Lambda_{26} = \Lambda_{30} = e^{-ik_2}$, $\Lambda_{29} = \Lambda_{33} = e^{-ik_3}$, $\Lambda_{32} = \Lambda_{36} = e^{-ik_4}$, $\Lambda_{35} = \Lambda_{39} = e^{ik_1}$, $\Lambda_{41} = \Lambda_{45} = e^{ik_2}$, $\Lambda_{44} = \Lambda_{48} = e^{ik_3}$, $\Lambda_{27} = \Lambda_{47} = e^{ik_4}$, and other diagonal elements are 1. By combining Eq.(5) and Eq.(6), we obtain the Bloch eigen-equation,

$$S(\mathbf{k})|b(\mathbf{k})\rangle = e^{-i\varphi(\mathbf{k})}|b(\mathbf{k})\rangle, \tag{7}$$

where $S(\mathbf{k}) = \Lambda^{-1}(\mathbf{k})S_{\text{unitcell}}$. As $S(\mathbf{k})$ is a unitary matrix, the eigenvalue $e^{-i\varphi(\mathbf{k})}$ is defined on the unit circle of the complex plane, which results in a real-valued phase $\varphi(\mathbf{k})$ lying in the region of $(0, 2\pi]$.

One can find that Eq.(7) takes a similar form to the Floquet eigen-equation of a periodically driven lattice, with the phase $\varphi(\mathbf{k})$ playing the role of quasi-energy, and $S(\mathbf{k})$ the one of the unitary Floquet time-evolution operators. To be more specific, the hyperbolic scattering network can be mapped into a cyclic oriented network describing a quantum walk, with $S(\mathbf{k})$ being the time-evolution operator of the system [2, 5–7], as shown in Supplementary Fig.2. Due to the non-reciprocity of the three-port scattering nodes in the original network (Supplementary Fig.2a), the links (Supplementary Fig.2b) are now unidirectional, which force wave packets traveling in this oriented graph to experience scattering on the four-link vertices (S_1, S_2, S_3) in a sequential order. This cyclicity plays the role of time-ordering in the associated stepwise time-dependent system, and allows both the restauration of the notion of time-modulation and the exact mapping to a time-Floquet system. The order is determined by the connection condition in the oriented network.

After forming the Bloch eigen-equation for our hyperbolic scattering network, we calculate the Floquet band structures to explore the hyperbolic topological physics. Supplementary Fig.3 shows the Floquet band structures when the angular parameters are $\xi = -\eta = \pi/8$ and $\xi = -\eta = \pi/4$, respectively. Each band structure has 48 bulk bands, since a unit cell has 16 circulators and 48 ports. Besides, one finds that both band structures exhibit degenerate bands, with different numbers of bandgaps, whose topology can be further classified by a Chern vector [8] and a properly defined homotopy invariant [5].

We also calculate the Floquet band structures at two typical points of the phase diagram, $\xi = -\eta = 0$ and $\xi = -\eta = \pi/6$, as shown in Supplementary Fig.4. The $\xi = -\eta = 0$ case relates to a hyperbolic reciprocal network consisting of reciprocal circulators. In such case, all bandgaps are closed, indicating a semi-metallic phase (see Supplementary Fig.4a).

The $\xi = -\eta = \pi/6$ case corresponds to a perfect circulator network with infinite isolation, featuring 48 flat bulk bands connected by dispersionless edge modes in all bandgaps, thus revealing an anomalous phase at the so-called phase-rotation symmetric point [5] (see Supplementary Fig.4b).

Supplementary Note 3: DOS comparisons between 1D hyperbolic band theory (HBT) and finite networks

Since the non-commutative Fuchsian group has higher-dimensional irreducible representations, it is important to explore the extent to which the 1D irreducible representations we have employed are able to capture a large amount of the bulk modes.

To address this question, we compare the total DOS obtained from HBT for a super cell and exact diagonalization for a finite network. We adopt the anomalous phase with angular parameters of $\xi = -\eta = \pi/8$ as an example. The DOS is defined as $\mathrm{DOS}(\varphi) = N(\varphi)/\delta(\varphi)$, where $N(\varphi)$ counts the number of eigenmodes within $[\varphi, \varphi + \delta(\varphi)]$. The computed DOS for a super cell (see Supplementary Fig.6a) is in agreement with that obtained from exact diagonalization for a closed finite network (see Supplementary Fig.6b), both of which clearly identify the edge modes and bulk modes. This distinction is further confirmed by the skin distance for a closed finite network and the two-port edge transmission on an open finite network, as shown in Supplementary Figs.6c-d, respectively.

Next, to test how well the 1D HBT describes the infinite lattice behavior, we compare the bulk DOS obtained from HBT for a unit cell, HBT for a super cell, and exact diagonalization for a finite network. In the case of the super cell and the finite network, the bulk DOS is determined by extracting the bulk modes using the skin distance, which effectively removes the boundary contribution to the total DOS. As shown in Supplementary Fig.7, the results show that a significant portion of the DOS remains in good agreement, giving us confidence that 1D HBT captures the bulk modes with sufficient accuracy in our hyperbolic scattering network.

Supplementary Note 4: Wilson-loop extraction of Chern vectors

In order to classify the bandgaps and to investigate the topological phases of the hyperbolic network, we calculate the first Chern numbers $C_{ij} \in \mathbb{N}$ for every degenerate bands in the six two-dimensional surface Brillouin zones (k_i, k_j), $i < j \in \mathbb{Z}\{1, ..., 4\}$. As our model possesses an eightfold rotational symmetry, the scattering matrices of the model, $S(k_1, k_2, k_3, k_4)$ and $S(k_2, k_3, k_4, -k_1)$, are linked by a unitary transformation. Consequently, the six first Chern numbers exhibit degeneracy such that only two are deemed independent, that is, $C_{12} = C_{23} = C_{34} = C_{14} = C_a$ and $C_{13} = C_{24} = C_b$. Accordingly, one could extend the six

first Chern numbers to a Chern vector $\mathbf{C} = (C_a, C_b)$ characterizing topological orders of the hyperbolic network in the four-dimensional momentum space.

We then calculate the Chern vector for each degenerate bands using the Wilson loop technique [9]. Supplementary Fig.8 shows the results for an anomalous phase ($\xi = -\eta = \pi/8$) and a Chern phase ($\xi = -\eta = \pi/4$). Note that, due to the chiral symmetry, bands at $\varphi \in [0, \pi]$ carry the same information as those at $\varphi \in [\pi, 2\pi]$. Thus, we here only plot the results of the bands at $\varphi \in [0, \pi]$, which are labelled from No.1 to No.24. One can find that the anomalous phase holds 4 sixfold degenerate bands with vanishing Chern vectors; yet the Chern phase possesses 3 sixfold degenerate bands with zero Chern vectors, and 2 threefold degenerate bands with non-zero Chern vectors (i.e., bands No.7-9 and No.10-12).

Supplementary Note 5: Topological invariant based on Laughlin's pump argument

In this section, we propose an effective approach to tackle the topological invariant of hyperbolic lattices by generalizing the Laughlin's pump argument. This provides us a way to directly measure topological invariant in our hyperbolic scattering network, and is applicable for arbitrary unitary scattering networks.

In the Laughlin's pump model [10–12], a ribbon is wrapped up into a cylinder. When applying an additional adiabatic-driven magnetic flux through the cylinder, electrons are pumped from one edge of the cylinder to the other, whose number per adiabatic-driven cycle yields the topological invariant. Inspired by this idea, we design a Corbino disk sample (see Fig.3a in the main text) to experimentally measure the topological invariant for the hyperbolic scattering network [13]. We first cut the sample in radial direction, and second bridge the gap by imparting the twisted boundary condition with a non-reciprocal phase $\pm\Phi$, which takes the role of Laughlin's magnetic flux (see Fig.3b in the main text). When the non-reciprocal phase equals zero, the twisted boundary condition behaves like a Bloch periodic boundary condition, which forms a discrete set of points on the continuous band structure of the Corbino disk sample (infinite in circular direction). When adiabatically increasing the non-reciprocal phase from 0 to 2π, the discrete quasi-energy points shift in one direction, and finally replace their neighbors on the right. It means that during a cycle, each Bloch mode physically allowed at a particular value of quasi-energy φ will yield a resonance in this finite system, at least once during the cycle. These resonances can be detected from a simple edge probe reflection measurement, since internal resonances always translate to a particular value of the scattering phase, e.g. π, if the direct reflection phase is zero (this exact value does not matter). In a topological band gap, a chiral edge mode contributes to a single crossing with the resonance condition, which is topological and cannot be eliminated, thus the edge probe reflection coefficient is forced to wind on the

complex plane. On the contrary, bulk modes (or any other non-topological modes) must always cross the resonance condition an even number of times without winding.

Taking the hyperbolic scattering network at $\xi = -\eta = 7\pi/24$ as an example, whose ribbon band structure is shown in Supplementary Fig.10a, the winding number W is computed at $\varphi = \pi/10$ (an edge mode), $\varphi = \pi/4$ (a bulk mode), and $\varphi = \pi/2$ (a mode in a trivial bandgap), as shown in Supplementary Fig.10c. As expected, the reflection coefficient of an edge mode shows a non-contractible loop on the complex plane, while that of a bulk mode and a mode in a trivial bandgap do not yield any winding. We also numerically calculate the winding number W on $\xi = -\eta$ line (see Supplementary Fig.10b), which is consistent to the bandgap maps in Supplementary Fig.9, despite a small mismatch due to the finite size of the sample.

Supplementary Note 6: Robustness of hyperbolic anomalous and Chern phases

In the 2D Euclidean space, it was shown that in the anomalous phase, trivial Anderson localization can surprisingly be completely avoided, whereas Chern phases behave as expected and trivially localize [2, 7]. The question we address here is: does this behavior remain true in hyperbolic cases?

In this section, we perform the quantitative studies of robustness of two hyperbolic phases. We consider a phase-disordered hyperbolic scattering network, where the disorder is introduced by adding randomly generated phase delays within $[-\delta\varphi/2, \delta\varphi/2]$ around $\varphi = \pi/8$, varying $\delta\varphi$. Each value on the solid line is averaged over 500 realizations of random disorder. Supplementary Fig.11a shows the average transmission between two ports on the boundary of the disordered network, with the dashed lines represents the first and last quartiles (Q1 and Q3). Remarkably, the chiral edge modes in the anomalous phase ($\xi = -\eta = \pi/8$) could survive under strong disorder strength up to the maximal value of 2π, in stark contrast to those in the Chern phase ($\xi = -\eta = 7\pi/24$), extending a result already known in Euclidean honeycomb lattices [2, 7]. One reason is that phase disorder in the anomalous phase introduces only fluctuations of quasi-energy that can locally switch the material between a bulk band and a topological gap, whereas a disordered Chern will be gradually "doped" with pieces of trivial insulators, eventually localizing every modes including the edge modes. Besides, we compute the average transmission along the edge in the phase-disordered network by varying $\xi = -\eta$ and disordered strength $\delta\varphi$, as shown in Supplementary Fig.11b. This statistical analysis provides further evidence of the existence of an anomalous disordered phase, since a region of the parameters space remains blue regardless of disorder. Finally, we compute the average transmission in the presence of fully-random phase disorder (with a disorder strength of 2π) for any possible circulator

(Supplementary Fig.11c). We confirm that a region of high average transmission persists in the anomalous phase, clearly evidencing the possibility for anomalous hyperbolic networks to survive arbitrarily large distributed phase disorder.

Supplementary Note 7: Design

The hyperbolic non-reciprocal scattering wave network consists of 200 circulators soldered on a printed circuit board (PCB). The PCB is a 0.508 mm thick Taconic TLY-5 laminate (dielectric loss $\tan\delta = 0.0009$ at 10 GHz) with 1 oz copper layers on the top and bottom sides. The circulator (UIYSC9B55T6, UIY Co.) is composed of three ports placed 120° apart from each other. It contains the ferrite disks and magnets inside, which enable the non-reciprocity. That is, without the magnetic field, the circulator would work in a reciprocal way, and support two degenerate modes, left-handed and right-handed modes, at $\omega_+ = \omega_- = \omega_0$. To bias it, the magnets provide a magnetic field of 50 kAm^{-1} = 625 Oe, which polarizes the ferrite, and lifts the mode degeneracy to $\omega_+ \neq \omega_-$.

We adopt the two-layer grounded coplanar waveguide (CPW) as the phase link connecting the circulators, which can be flexibly bent, and is probably the most common solution for high frequency layout and routing. We pattern the CPWs on the top and bottom layers of the PCB, where the strips (grounds) on two layers are connected via metallic holes, as shown in Supplementary Fig.13a. There are several advantages to using a CPW design over a microstrip design. One is the high field isolation thanks to the surrounding ground plane, which provides additional electromagnetic interference shielding (see the field map in Supplementary Fig.13b). The second advantage is the low loss. As most of the field is confined above the dielectric substrate, waves would not experience much loss of the substrate. Supplementary Figs.13c-d plot the scattering properties of the designed CPW, showing a nearly 0 dB transmission, a relatively low reflection and a linear phase response. The third advantage is the high flexibility. Apart from the width of the strip s, there is an additional degree of freedom: the width of the gap between strip and ground w. For example, one can modify the impedance by tuning both s and w. Here, we set $s = 1.45$ mm and $w = 0.8$ mm, so that the impedance at ports 1 and 2 is around 50 Ohm (see Supplementary Fig.13e), and can match well with 50 Ohm coaxial probes for measurements. Since the CPW is known to behave as a pure phase delay φ in our operational frequency range, we model its phase as $\varphi = -\frac{2\pi f}{c}\sqrt{\varepsilon_{eff}}L$, and the transmission as $T = e^{\varphi\frac{\tan\sigma}{2}}$, where f is the operational frequency, c is the speed of light, L is the length of CPW, $\tan\sigma$ is the dissipation loss tangent, ε_{eff} is the effective permittivity that could be obtained according to a well-known empirical formula.

We also design a reconfigurable non-reciprocal phase shifter to construct the twisted boundary condition for the topological invariant measurement. As shown in Supplementary

Fig.14a, it consists of two identical and reciprocal phase shifters on the right and left sides, which are connected at the two junction points by two circulators. The circulators provides the non-reciprocity for the whole structure. Each reciprocal phase shifter is composed of three parts: two reflective loads, a 90-degree hybrid coupler and two high pass filters. The reflective loads mainly contribute to continuously shift the value of the non-reciprocal phase, by tuning the capacitance of the varactors using external static voltages. The radial stubs serve as low-pass filters to isolate the microwave signal from the static voltage. The 90-degree hybrid coupler is used for wide-band impedance matching. The high pass filters isolate the static voltage V_1 from V_2 on two sides. All the components are built on a 1.016 mm thick Rogers RO4350B laminate with 1 oz copper layers on the top and bottom sides. The varactors are SMV2020-079LF (Skywork Co.), namely silicon hyperabrupt junction varactor diodes. The measured phase values in Supplementary Fig.14b show that, by tuning the static voltages from 0 to 15 V, the non-reciprocal phase shifter exhibits a phase-shift range larger than 2π over a wide frequency range. In our topological invariant measurement, we fabricate three identical reconfigurable non-reciprocal phase shifters.

Supplementary Note 8: Measured scattering matrix of a circulator

Based on the CPW model, one can extract the scattering matrix of a single circulator by de-embedding the effect of the CPW. The resulting transmission, reflection and isolation of a single circulator are displayed in Supplementary Fig.12a, confirming the C_3 symmetry of the scattering matrix in the considered frequency range. We also check the unitarity of the measured scattering matrix. Supplementary Fig.12b plots the eigenvalues of the measured scattering matrix on the complex plane, which mostly locate on a unit circle, consistent with the unitarity assumption. Besides, we check the transmission phase consistency among different circulators. To do so, we define a standard deviation for the circulators as,

$$\sigma = \left[\frac{1}{N}\sum_{i=1}^{N} |\phi_{21}^i - \bar{\phi}|^2 + |\phi_{32}^i - \bar{\phi}|^2 + |\phi_{13}^i - \bar{\phi}|^2\right]^{1/2}, \tag{8}$$

where ϕ_{21}, ϕ_{32} and ϕ_{13} are the transmission phases, and N is the number of circulators. The results are plotted in Supplementary Fig.12c. We see that this quantity is below 9° at all frequencies, which is good enough.

Additionally, by employing the frequency dispersions of the CPW and the circulator, we determine the practical bandgap map, which is used for design purposes. This bandgap map is a deformed version plotted as a function of the operational frequency f and length of CPWs L, as shown in Supplementary Fig.12d. One can find a topological phase transition happens at around 5.5 GHz, where the anomalous phase stands in $[5.5, 6.5]$ GHz with low

reflection of the circulators, and the Chern phase is in $[5, 5.5]$ GHz with high reflection. With the aid of the map, we adopt $L_1 = 57$ mm for eight CPWs on the central octagon of the hyperbolic network sample, and $L_2 = 21$ mm for the remaining CPWs. Compared to the shorter CPW, the longer CPW is designed to have an additional 2π phase delay at 5.93 GHz, with a very small phase difference ($\leq 5\%$) within $[5.75, 6.10]$ GHz.

Supplementary Note 9: Measurements

For the transmission measurement, we use a vector network analyzer (VNA; ZNB20, R&S) to collect the transmission amplitude and phase between two boundary ports (see Supplementary Fig.15a). The remaining boundary ports are perfectly matched with 50 Ohm terminations (full reflection). For the field map measurement, one port of VNA is connected to a signal input port on the sample. Another port of VNA is connected to a coaxial probe, which is used to manually measure the fields at middle of the CPWs (see Supplementary Fig.15b). For the topological invariant measurement, the CPWs between the ports on the red-dashed boxes in Supplementary Fig.15a are disconnected. Instead, these ports are linked in pairs by three phase shifters, which provide the non-reciprocal phases $\pm\Phi$ and form twisted boundary conditions. The non-reciprocal phases are controlled by voltages from two DC sources. We then record the scattering parameters of the hyperbolic network for each value of $\Phi \in [0, 2\pi]$, to further analyze the topological invariant.

In order to achieve all the measurements on the same sample, we design three small pieces of PCBs, termed as S2s (see Supplementary Fig.15c for a S2). For the transmission and field map measurements, the top layers of S2s are attached on the bottom layer of the hyperbolic sample (the regions marked by red-dashed boxes in Supplementary Fig.15a; termed as S1s) by screws, behaving as proper phase delay lines (CPWs). For the topological invariant measurement, S2s are removed, so that the CPWs are disconnected. The surrounding ports are then linked in pairs by three phase shifters.

It should be noted that due to the negative spatial curvature, the boundary circulators occupy a large portion of the total circulators, regardless of the size of the hyperbolic lattice (see Supplementary Fig.1a). In our experimental design, we have 152 boundary circulators and 48 bulk circulators, leading to a lengthy boundary with a comparatively compact bulk area. In order to achieve highly efficient wave propagation along such a extensive boundary, we intentionally employ finely-tuned CPWs with strongly confined fields instead of microstrips. As a consequence, the anomalous edge modes, for example, at 5.8 GHz and 6.0 GHz shown in Fig.3e of the main text, are able to travel through over a quarter of the total boundary - that is, more than 50 circulators - with each boundary node causing a loss of only 0.6 dB.

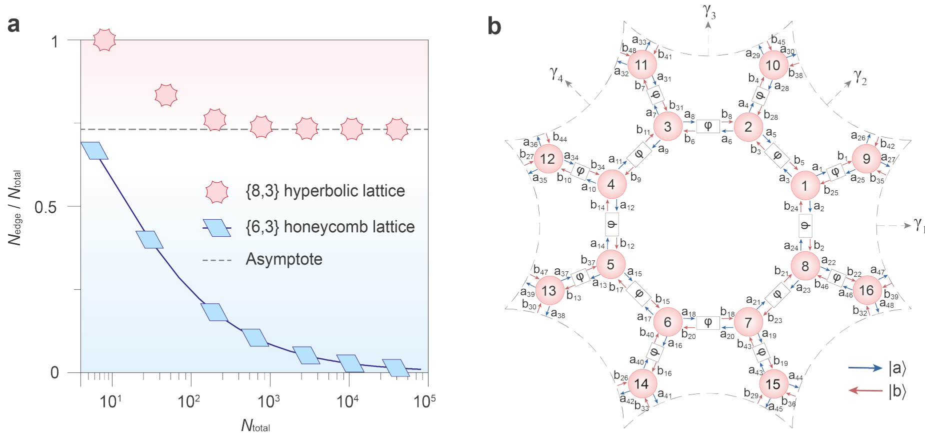

Supplementary Fig. 1. Ratio of edge sites over total sites in Euclidean and hyperbolic lattices, and unit cell of $\{8, 3\}$ hyperbolic scattering network. a, The edge sites of the hyperbolic lattices (e.g., $\{8, 3\}$ hyperbolic lattice, red octagons) occupy a finite large portion due to the negative spatial curvature, and the ratio approaches a finite constant value in the thermodynamic limit. In contrast, the edge-site ratio in Euclidean lattices (e.g., $\{6, 3\}$ honeycomb lattice, blue rhombuses) vanishes. **b**, The unit cell of $\{8, 3\}$ hyperbolic network contains 16 circulators interconnected by bidirectional links with phase delay φ. Scattering waves in the unit cell are characterized by the state vectors $|a(\mathbf{k})\rangle$ and $|b(\mathbf{k})\rangle$, each of which comprises 48 components. Four pairwise edges of the unit cell define four translation generators: $\gamma_\mu, \mu \in \{1, ..., 4\}$.

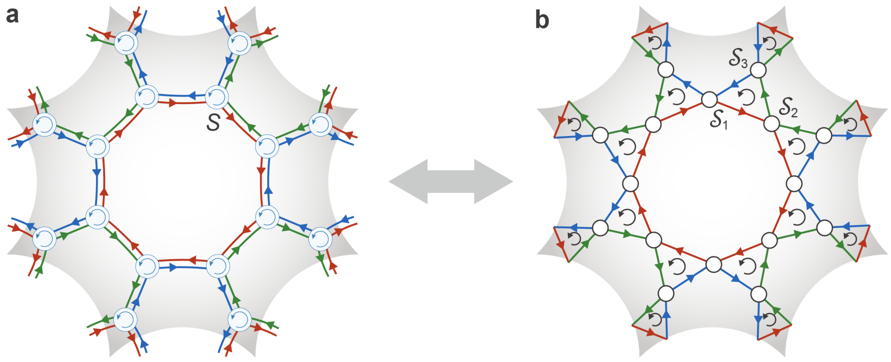

Supplementary Fig. 2. Mapping of the $\{8, 3\}$ hyperbolic lattice (panel a) to a cyclic oriented network (panel b).

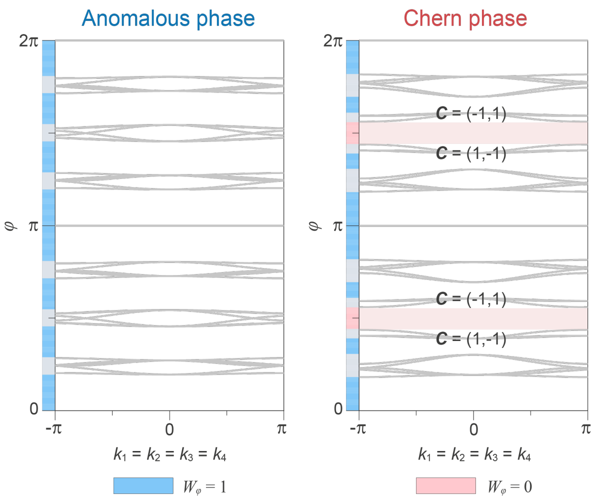

Supplementary Fig. 3. Floquet band structures of the unit cell. Floquet band structures computed on Eq.(7) along $k_1 = k_2 = k_3 = k_4$. The angular parameters of the circulators are $\xi = -\eta = \pi/8$ (left panel) and $\xi = -\eta = \pi/4$ (right panel), respectively, which correspond to the hyperbolic version of anomalous Floquet and Chern insulators. For the anomalous Floquet insulator, the unitary homotopy invariants for all bandgaps are $W_\varphi = 1$ (blue areas on the left side of band structures), with the vanishing Chern vectors $\mathbf{C} = (C_a, C_b) = \mathbf{0}$ for all sixfold degenerate bands. For the Chern insulator, there are two additional trivial bandgaps with $W_\varphi = 0$ (red areas), surrounded by bands with non-zero Chern vectors.

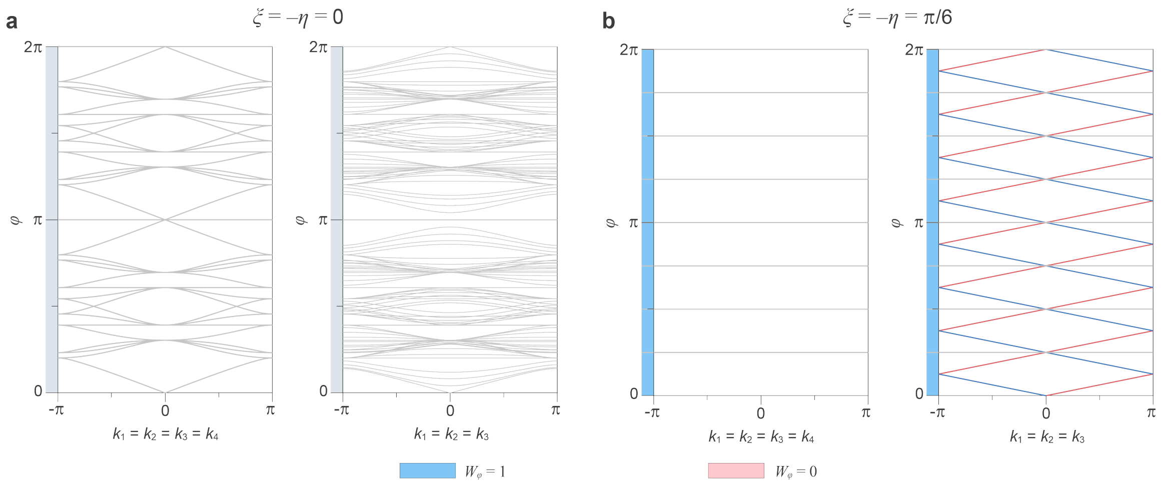

Supplementary Fig. 4. Floquet band structures of two special cases. **a**, Floquet band structures of a unit cell (left panel) and of a super cell (right panel), with the circulator angular parameters being $\xi = -\eta = 0$. The blue and red areas on the left side of the band structures characterize the topological bandgaps with the homotopy invariant $W_\varphi = 1$ and trivial bandgaps with $W_\varphi = 0$, respectively. This case corresponds to a hyperbolic reciprocal network with all bandgaps closed, indicating the phase transition point among trivial phase, Chern phase and anomalous phase. **b**, Same as panel a, but when $\xi = -\eta = \pi/6$. This case relates to a phase-rotation symmetric network of perfect matched circulators, featuring flat bulk bands with dispersionless edge modes, which can only occur at the anomalous phase.

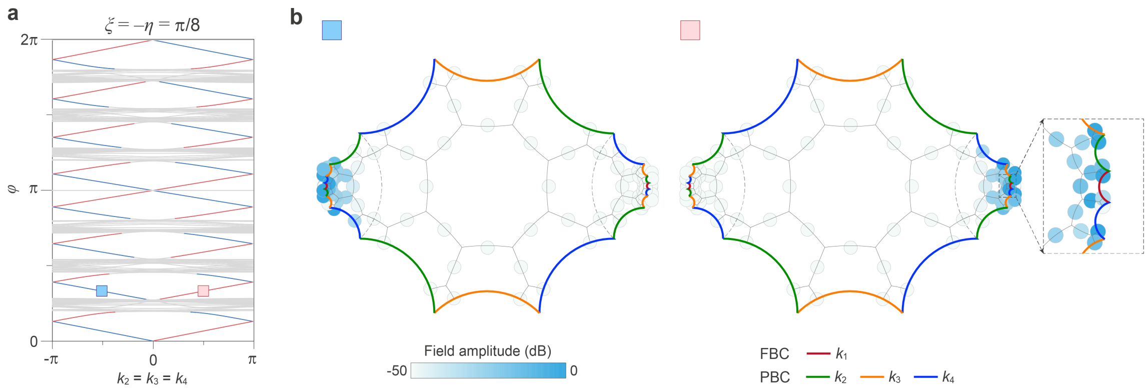

Supplementary Fig. 5. Super cell with the profiles of chiral edge modes. **a**, Floquet band structure of a super cell, with the circulator angular parameters being $\xi = -\eta = \pi/8$. **b**, Profiles of chiral edge modes, corresponding to markers in **a**. The super cell contains three unit cells (split by the dashed lines), where the periodic boundary conditions (PBCs, green, orange and blue lines) are employed along the k_2, k_3, and k_4 directions, and the full-reflection boundary conditions (FBCs, red lines) are applied along k_1.

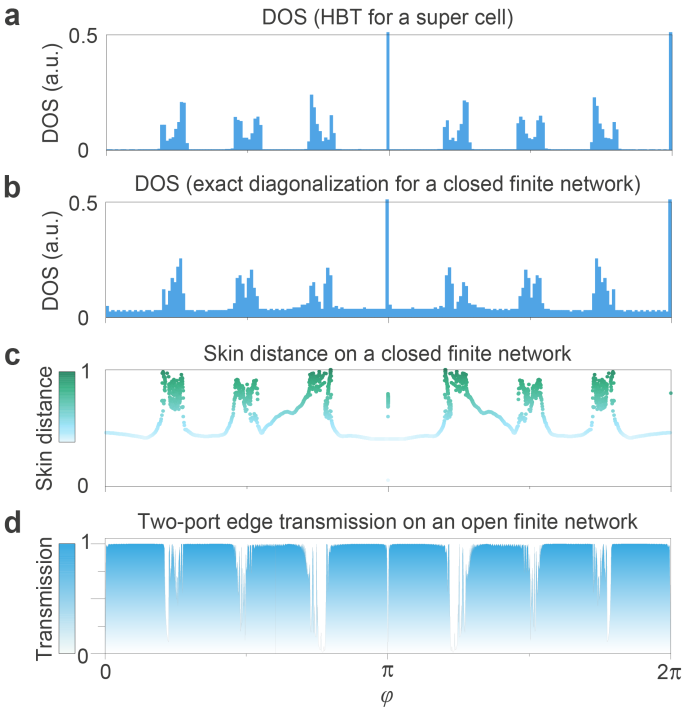

Supplementary Fig. 6. Identification of chiral edge modes and bulk modes. **a**, DOS obtained from HBT for a super cell. The super cell consists of 5 unit cells, with the periodic boundary conditions along k_2, k_3, and k_4 directions, and the full-reflection boundary conditions along k_1. **b**, DOS obtained from the exact diagonalization for a closed 4-layer network. **c**, Skin distance for a closed 4-layer network. The skin distance is defined as $p_s(|b\rangle) = \sum_i p_s(i)|b_i|^2 / \sum_i |b_i|^2$, where $|b_i|$ is the amplitude of mode $|b\rangle$ at port i, and $p_s(i) \in \mathbb{N}$ is the skin index of port i, ranging from $p_s = 1$ on the boundary to a (size-dependent) maximal value at the center. **d**, Two-port edge transmissions on an open 4-layer network. Here, we take the anomalous phase ($\xi = -\eta = \pi/8$) as an example.

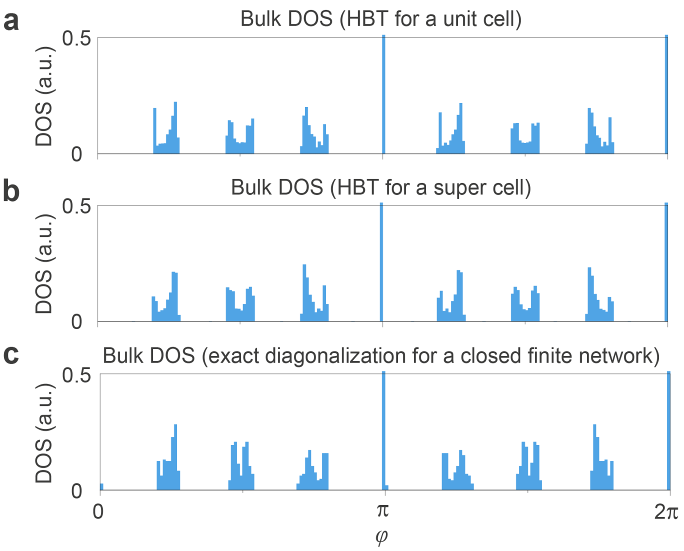

Supplementary Fig. 7. Bulk DOS comparison. a, Bulk DOS obtain from HBT for a unit cell. **b-c** Bulk DOS obtain from HBT for a super cell (panel b) and exact diagonalization for a closed 4-layer network (panel c). The bulk DOS is determined by extracting the bulk modes using the skin distance with a threshold of 0.7.

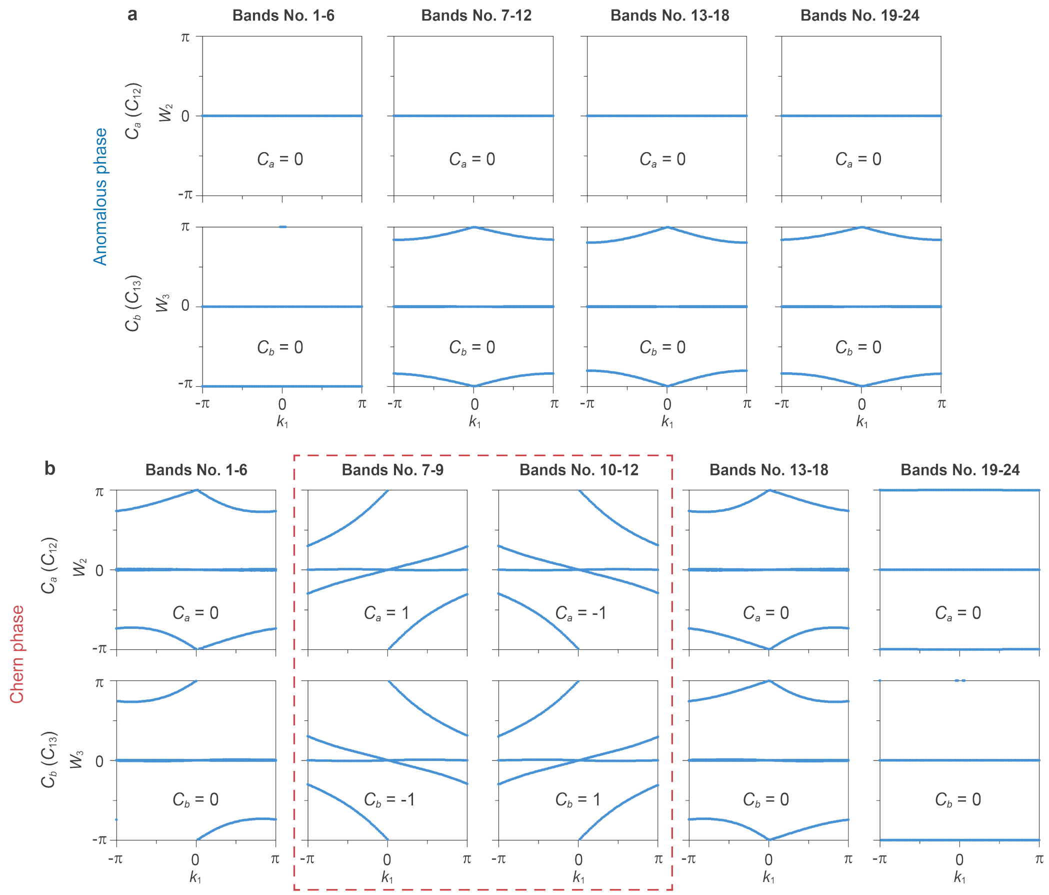

Supplementary Fig. 8. Wilson-loop extraction of Chern vectors. The Chern vector $\mathbf{C} = (C_a, C_b)$ is computed based on Wilson-loop technique for every degenerate bands at $\xi = -\eta = \pi/8$ (panel a, anomalous phase) and $\xi = -\eta = \pi/4$ (panel b, Chern phase). Here, we only consider the bands at $\varphi \in [0, \pi]$, which are labelled from No.1 to No.24.

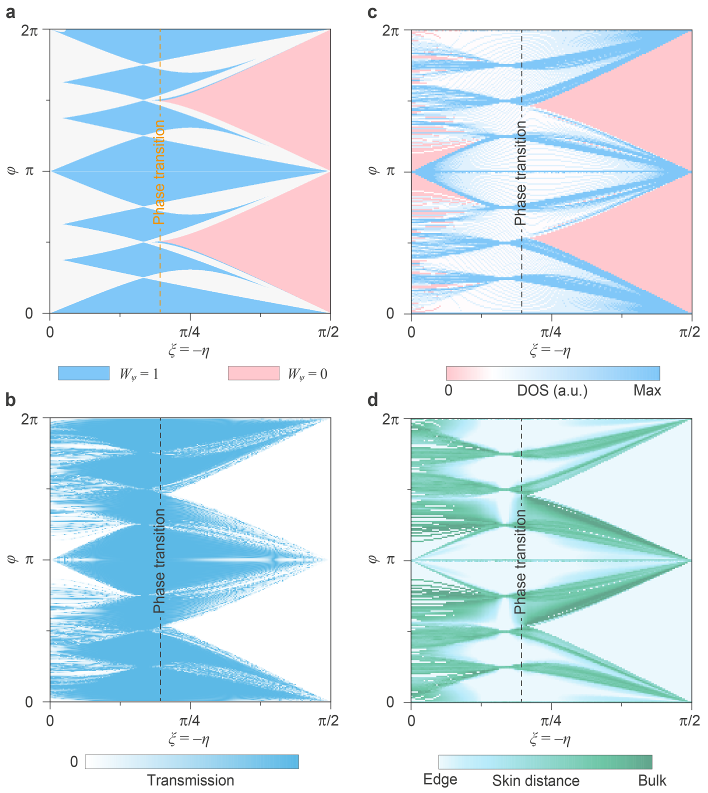

Supplementary Fig. 9. Topological bandgap maps on $\xi = -\eta$ line. We unveil the topological bandgap maps by computing the homotopy invariant W_φ in the thermodynamic limit (panel a), edge transmission for a finite network (panel b), density of states (DOS) and skin distance for a closed finite network (panels c and d). One can find these bandgap maps are consistent with each other.

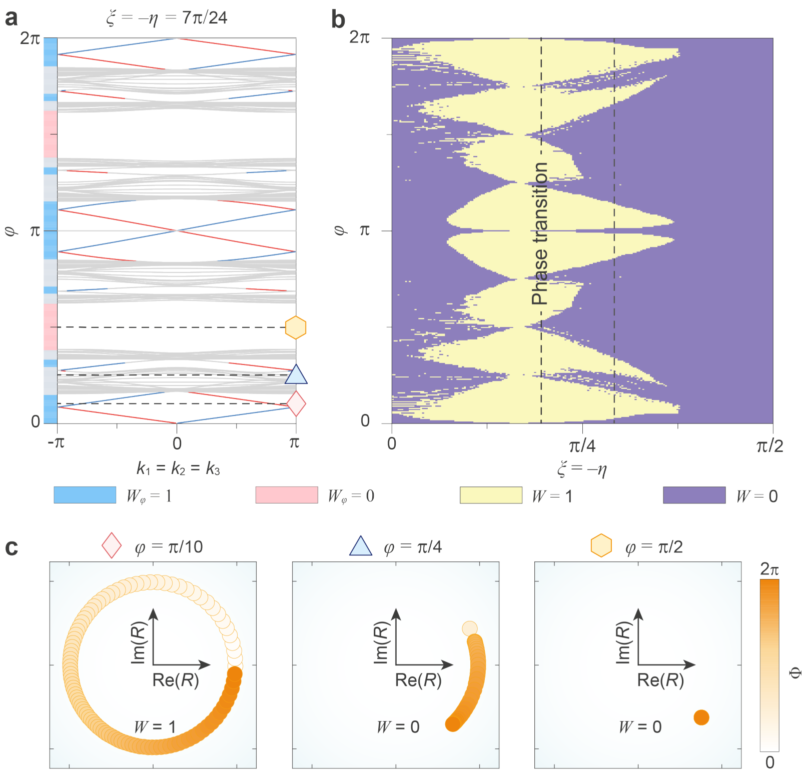

Supplementary Fig. 10. Topological invariant in the hyperbolic scattering network. a, Floquet band structure of a super cell at $\xi = -\eta = 7\pi/24$. **b**, Numerically-calculated topological invariant W as a function of $\xi = -\eta$, which is consistent to the bandgap maps in Supplementary Fig.9. **c**, Windings of the reflection coefficient on the complex plane when quasi-energy $\varphi = \pi/10$ (an edge mode), $\varphi = \pi/4$ (a bulk mode), and $\varphi = \pi/2$ (a mode in a trivial bandgap). As expected, the reflection coefficient of the edge mode winds in a non-contractible loop, while that of the bulk mode and the mode in the trivial bandgap does not exhibit any winding.

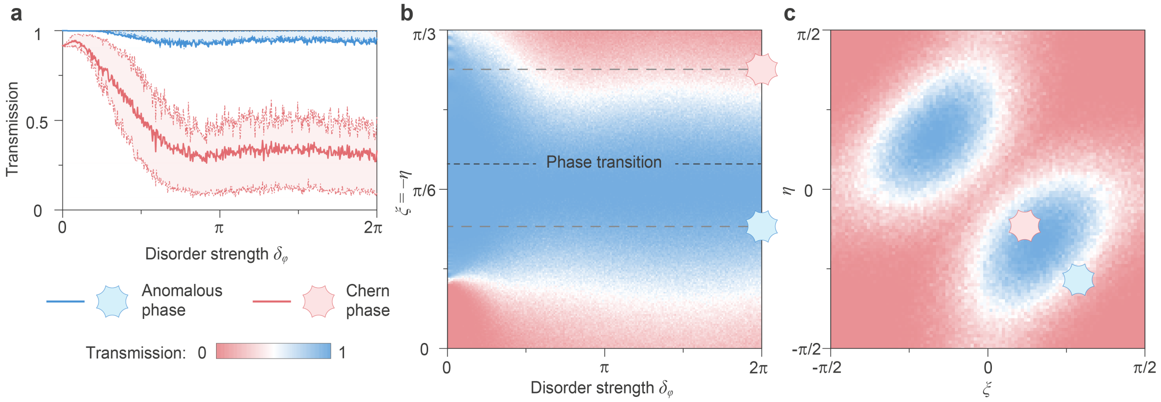

Supplementary Fig. 11. Robustness of hyperbolic anomalous and Chern phases. a, Transmission between two ports on the boundary of a phase-disordered hyperbolic scattering network. The disorder is introduced by adding randomly generated phase delays within $[-\delta\varphi/2, \delta\varphi/2]$ around $\varphi = \pi/8$, varying $\delta\varphi$. Here, anomalous and Chern phases have the angular parameters of $\xi = -\eta = \pi/8$ and $\xi = -\eta = 7\pi/24$, respectively. Each value on the solid line is averaged over 500 realizations of random disorder. The dashed lines are the first and last quartiles (Q1 and Q3). **b**, Average transmission in the phase-disordered network as a function of $\xi = -\eta$ and disordered strength $\delta\varphi$. **c**, Average transmission in the presence of fully-random phase disorder (with a disorder strength of 2π) at each point of the phase diagram.

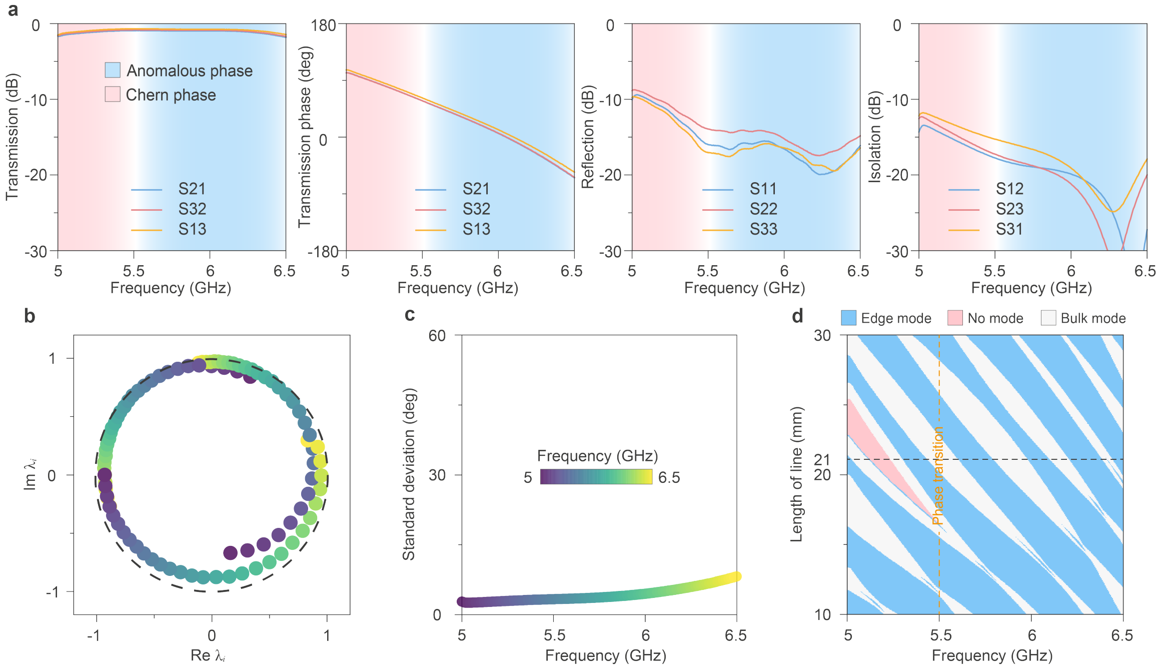

Supplementary Fig. 12. Measured scattering properties of a single circulator. **a**, Measured transmission, reflection and isolation of a single circulator, indicating C_3 symmetry of the scattering matrix. The blue-shaded (red-shaded) area represents the anomalous (Chern) phase. The topological transition happens at around 5.5 GHz. **b**, Eigenvalues of the measured scattering matrix lying on a unit circle, showing nearly-unitary behavior over the entire experimental bandwidth. **c**, Standard deviation of transmission phases among circulator samples, which is smaller than 9° at our considered frequencies. **d**, Predicted bandgap map as a function of length of CPW L and operational frequency f. Black-dashed vertical line marks the topological phase transition point.

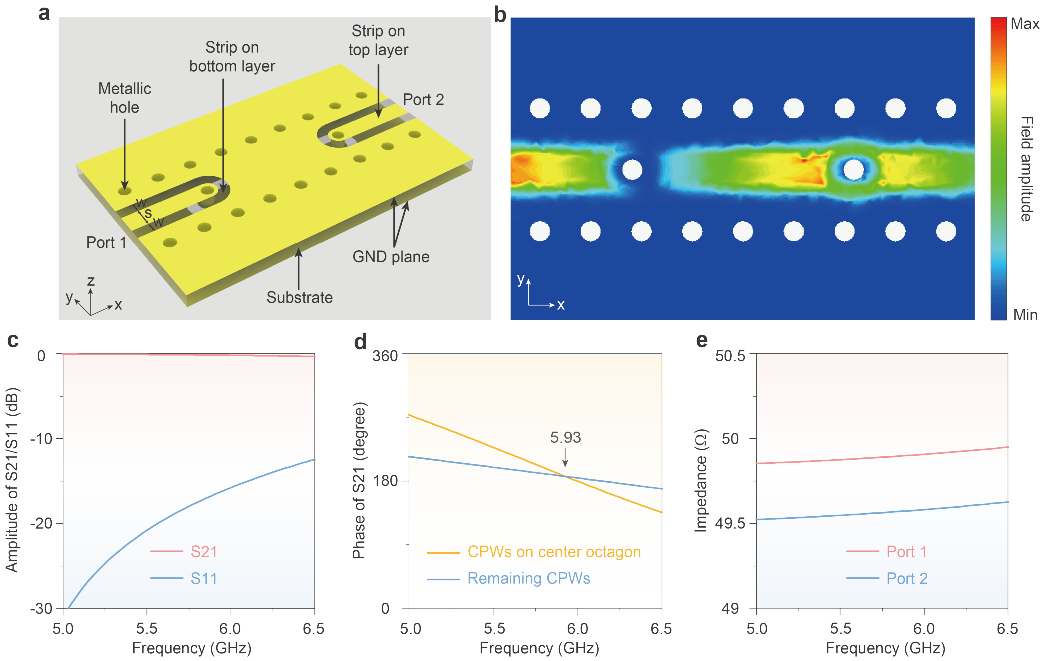

Supplementary Fig. 13. Coplanar waveguide (CPW) serves as a phase link. In the experiments, we adopt a two-layer grounded CPW as a phase link connecting the circulators. **a**, Schematic of a two-layer grounded CPW, where the strips (grounds) on top and bottom layers are connected via metallic holes, sandwiching a 0.508 mm thick dielectric laminate (Taconic TLY-5). **b**, Simulated field distribution (input at port 1) on the middle layer at 5.75 GHz, showing high field isolation. **c**, High transmission and low reflection of the CPW with length $L_2 = 21$ mm. **d**, Linear phase response of the CPW with length $L_1 = 57$ mm (orange line) and $L_2 = 21$ mm (blue line), respectively. The longer CPW has an additional 360° phase delay than the shorter one at 5.93 GHz, with a very small phase difference ($\leq 5\%$) within $[5.75, 6.10]$ GHz. **e**, Impedances at two ports of the CPW. Here, we set $s = 1.45$ mm and $w = 0.8$ mm, so that the impedance is around 50 Ohm, and can match well with other components and 50 Ohm coaxial probes for measurements.

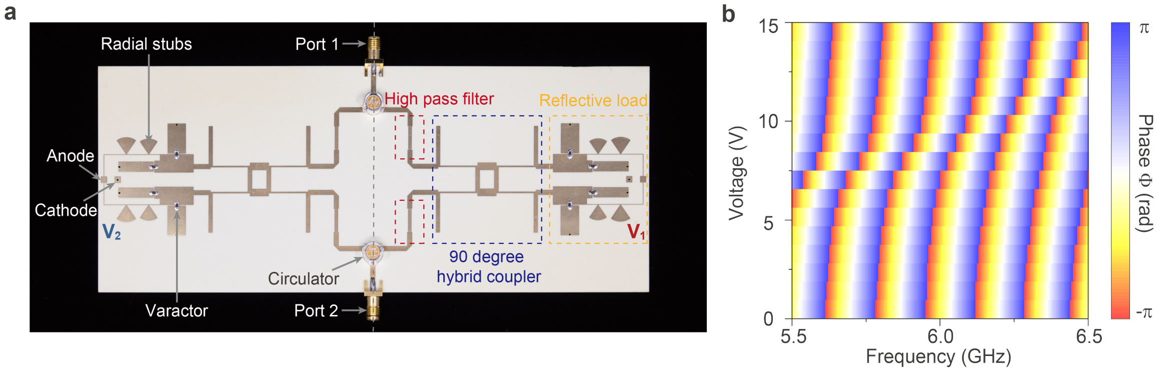

Supplementary Fig. 14. Design of a reconfigurable non-reciprocal phase shifter. a, Photograph of a reconfigurable non-reciprocal phase shifter, consisting of two identical and reciprocal phase shifters on two sides. The wave paths in these two phase shifters are separated by two circulators for independent phase controls. Each reciprocal phase shifter is composed of three parts: two reflective loads, a 90-degree hybrid coupler and two high pass filters. **b**, Measured phase value as a function of static voltage V and operational frequency f. One can find the non-reciprocal phase shifter exhibits a phase-shift range larger than 2π over a wide band.

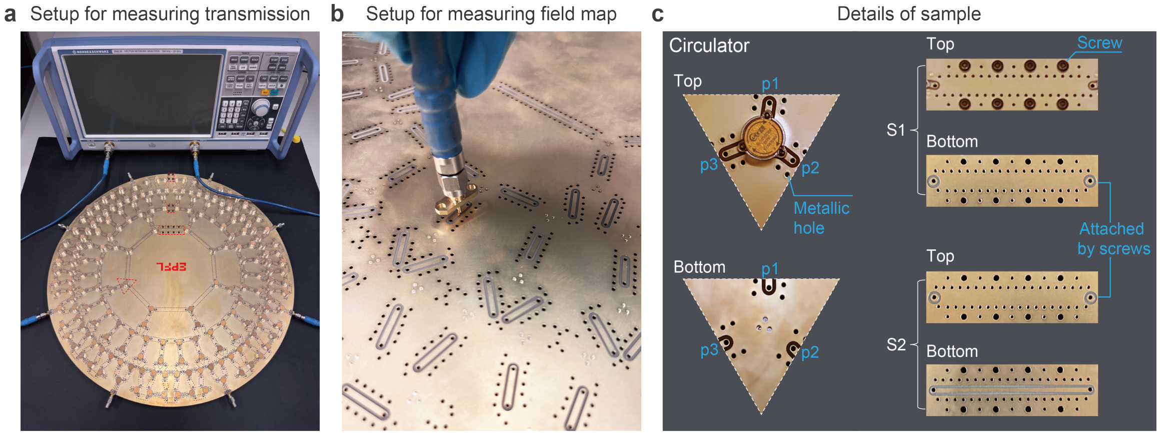

Supplementary Fig. 15. Experimental setups. **a**, Setup for the transmission measurement, consisting of a vector network analyzer (VNA; ZNB20, R&S), and two cables connecting VNA and two boundary ports of the sample. **b**, Setup for the field map measurement with a coaxial probe for measuring fields at middle of CPWs. **c**, Details of the sample. Left panel (corresponding to the red-dashed triangle in panel a): the circulators are attached on the top layer of the PCB. Right panel: for the transmission and field map measurements, the top layer of the designed S2 is attached on the bottom layer of S1 (a part of the PCB, marked by a red-dashed box in panel a) by screws, serving as a proper phase delay link; for the topological invariant measurement, S2 is removed.

References

[1] Khanikaev, A. B., Fleury, R., Mousavi, S. H., and Alù, A. Topologically robust sound propagation in an angular-momentum-biased graphene-like resonator lattice. *Nat. Commun.* **6**, 8260 (2015).

[2] Zhang, Z., Delplace, P., and Fleury, R. Superior robustness of anomalous non-reciprocal topological edge states. *Nature* **598**, 293–297 (2021).

[3] Fleury, R., Sounas, D. L., Sieck, C. F., Haberman, M. R., and Alù, A. Sound isolation and giant linear nonreciprocity in a compact acoustic circulator. *Science* **343**, 516–519 (2014).

[4] Boettcher, I., Gorshkov, A. V., Kollár, A. J., Maciejko, J., Rayan, S., and Thomale, R. Crystallography of hyperbolic lattices. *Phys. Rev. B* **105**, 125118 (2022).

[5] Delplace, P., Fruchart, M., and Tauber, C. Phase rotation symmetry and the topology of oriented scattering networks. *Phys. Rev. B* **95**, 205413 (2017).

[6] Delplace, P. Topological chiral modes in random scattering networks. *SciPost Phys.* **8**, 081 (2020).

[7] Zhang, Z., Delplace, P., and Fleury, R. Anomalous topological waves in strongly amorphous scattering networks. *Sci. Adv.* **9**, eadg318 (2023).

[8] Haldane, F. D. M. Berry curvature on the Fermi surface: anomalous Hall effect as a topological Fermi-liquid property. *Phys. Rev. Lett.* **93**, 206602 (2004).

[9] Wang, H.-X., Guo, G.-Y., and Jiang, J.-H. Band topology in classical waves: Wilson-loop approach to topological numbers and fragile topology. *New J. Phys.* **21**, 093029 (2019).

[10] Laughlin, R. B. Quantized Hall conductivity in two dimensions. *Phys. Rev. B* **23**, 5632–5633 (1981).

[11] Fabre, A., Bouhiron, J.-B., Satoor, T., Lopes, R., and Nascimbene, S. Laughlin's topological charge pump in an atomic Hall cylinder. *Phys. Rev. Lett.* **128**, 173202 (2022).

[12] Bardyn, C.-E., Filippone, M., and Giamarchi, T. Bulk pumping in two-dimensional topological phases. *Phys. Rev. B* **99**, 035150 (2019).

[13] Kawamura, M., Mogi, M., Yoshimi, R., Morimoto, T., Takahashi, K. S., Tsukazaki, A., Nagaosa, N., Kawasaki, M., and Tokura, Y. Laughlin charge pumping in a quantum anomalous Hall insulator. *Nat. Phys.* **19**, 333–337 (2023).
